# Author Correction: Para-infectious brain injury in COVID-19 persists at follow-up despite attenuated cytokine and autoantibody responses

**DOI:** 10.1038/s41467-024-47320-6

**Published:** 2024-04-04

**Authors:** Benedict D. Michael, Cordelia Dunai, Edward J. Needham, Kukatharmini Tharmaratnam, Robyn Williams, Yun Huang, Sarah A. Boardman, Jordan J. Clark, Parul Sharma, Krishanthi Subramaniam, Greta K. Wood, Ceryce Collie, Richard Digby, Alexander Ren, Emma Norton, Maya Leibowitz, Soraya Ebrahimi, Andrew Fower, Hannah Fox, Esteban Tato, Mark A. Ellul, Geraint Sunderland, Marie Held, Claire Hetherington, Franklyn N. Egbe, Alish Palmos, Kathy Stirrups, Alexander Grundmann, Anne-Cecile Chiollaz, Jean-Charles Sanchez, James P. Stewart, Michael Griffiths, Tom Solomon, Gerome Breen, Alasdair J. Coles, Nathalie Kingston, John R. Bradley, Patrick F. Chinnery, Jonathan Cavanagh, Sarosh R. Irani, Angela Vincent, J. Kenneth Baillie, Peter J. Openshaw, Malcolm G. Semple, ISARIC4C Investigators, J. Kenneth Baillie, Peter J. Openshaw, Malcolm G. Semple, Beatrice Alex, Petros Andrikopoulos, Benjamin Bach, Wendy S. Barclay, Debby Bogaert, Meera Chand, Kanta Chechi, Graham S. Cooke, Ana da Silva, Thushan de Silva, Annemarie B. Docherty, Gonçalo dos Santos, Marc-Emmanuel Dumas, Jake Dunning, Tom Fletcher, Christoper A. Green, William Greenhalf, Julian L. Griffin, Rishi K. Gupta, Ewen M. Harrison, Antonia Y. Wai, Karl Holden, Peter W. Horby, Samreen Ijaz, Saye Khoo, Paul Klenerman, Andrew Law, Matthew R. Lewis, Sonia Liggi, Wei S. Lim, Lynn Maslen, Alexander J. Mentzer, Laura Merson, Alison M. Meynert, Shona C. Moore, Mahdad Noursadeghi, Michael Olanipekun, Anthonia Osagie, Massimo Palmarini, Carlo Palmieri, William A. Paxton, Georgios Pollakis, Nicholas Price, Andrew Rambaut, David L. Robertson, Clark D. Russell, Vanessa Sancho-Shimizu, Caroline J. Sands, Janet T. Scott, Louise Sigfrid, Tom Solomon, Shiranee Sriskandan, David Stuart, Charlotte Summers, Olivia V. Swann, Zoltan Takats, Panteleimon Takis, Richard S. Tedder, A. A. R. Thompson, Emma C. Thomson, Ryan S. Thwaites, Lance C. Turtle, Maria Zambon, Thomas M. Drake, Cameron J. Fairfield, Stephen R. Knight, Kenneth A. Mclean, Derek Murphy, Lisa Norman, Riinu Pius, Catherine A. Shaw, Marie Connor, Jo Dalton, Carrol Gamble, Michelle Girvan, Sophie Halpin, Janet Harrison, Clare Jackson, James Lee, Laura Marsh, Daniel Plotkin, Stephanie Roberts, Egle Saviciute, Sara Clohisey, Ross Hendry, Susan Knight, Eva Lahnsteiner, Gary Leeming, Lucy Norris, James Scott-Brown, Sarah Tait, Murray Wham, Richard Clark, Audrey Coutts, Lorna Donnelly, Angie Fawkes, Tammy Gilchrist, Katarzyna Hafezi, Louise MacGillivray, Alan Maclean, Sarah McCafferty, Kirstie Morrice, Lee Murphy, Nicola Wrobel, Gail Carson, Kayode Adeniji, Daniel Agranoff, Ken Agwuh, Dhiraj Ail, Erin L. Aldera, Ana Alegria, Sam Allen, Brian Angus, Abdul Ashish, Dougal Atkinson, Shahedal Bari, Gavin Barlow, Stella Barnass, Nicholas Barrett, Christopher Bassford, Sneha Basude, David Baxter, Michael Beadsworth, Jolanta Bernatoniene, John Berridge, Colin Berry, Nicola Best, Pieter Bothma, Robin Brittain-Long, Naomi Bulteel, Tom Burden, Andrew Burtenshaw, Vikki Caruth, David Chadwick, Duncan Chambler, Nigel Chee, Jenny Child, Srikanth Chukkambotla, Tom Clark, Paul Collini, Catherine Cosgrove, Jason Cupitt, Maria-Teresa Cutino-Moguel, Paul Dark, Chris Dawson, Samir Dervisevic, Phil Donnison, Sam Douthwaite, Andrew Drummond, Ingrid DuRand, Ahilanadan Dushianthan, Tristan Dyer, Cariad Evans, Chi Eziefula, Chrisopher Fegan, Adam Finn, Duncan Fullerton, Sanjeev Garg, Atul Garg, Effrossyni Gkrania-Klotsas, Jo Godden, Arthur Goldsmith, Clive Graham, Tassos Grammatikopoulos, Elaine Hardy, Stuart Hartshorn, Daniel Harvey, Peter Havalda, Daniel B. Hawcutt, Maria Hobrok, Luke Hodgson, Anil Hormis, Joanne Howard, Michael Jacobs, Susan Jain, Paul Jennings, Agilan Kaliappan, Vidya Kasipandian, Stephen Kegg, Michael Kelsey, Jason Kendall, Caroline Kerrison, Ian Kerslake, Oliver Koch, Gouri Koduri, George Koshy, Shondipon Laha, Steven Laird, Susan Larkin, Tamas Leiner, Patrick Lillie, James Limb, Vanessa Linnett, Jeff Little, Mark Lyttle, Michael MacMahon, Emily MacNaughton, Ravish Mankregod, Huw Masson, Elijah Matovu, Katherine McCullough, Ruth McEwen, Manjula Meda, Gary Mills, Jane Minton, Kavya Mohandas, Quen Mok, James Moon, Elinoor Moore, Patrick Morgan, Craig Morris, Katherine Mortimore, Samuel Moses, Mbiye Mpenge, Rohinton Mulla, Michael Murphy, Thapas Nagarajan, Megan Nagel, Mark Nelson, Lillian Norris, Matthew K. O’Shea, Marlies Ostermann, Igor Otahal, Mark Pais, Selva Panchatsharam, Danai Papakonstantinou, Padmasayee Papineni, Hassan Paraiso, Brij Patel, Natalie Pattison, Justin Pepperell, Mark Peters, Mandeep Phull, Stefania Pintus, Tim Planche, Frank Post, David Price, Rachel Prout, Nikolas Rae, Henrik Reschreiter, Tim Reynolds, Neil Richardson, Mark Roberts, Devender Roberts, Alistair Rose, Guy Rousseau, Bobby Ruge, Brendan Ryan, Taranprit Saluja, Matthias L. Schmid, Aarti Shah, Manu Shankar-Hari, Prad Shanmuga, Anil Sharma, Anna Shawcross, Jagtur S. Pooni, Jeremy Sizer, Richard Smith, Catherine Snelson, Nick Spittle, Nikki Staines, Tom Stambach, Richard Stewart, Pradeep Subudhi, Tamas Szakmany, Kate Tatham, Jo Thomas, Chris Thompson, Robert Thompson, Ascanio Tridente, Darell Tupper-Carey, Mary Twagira, Nick Vallotton, Rama Vancheeswaran, Rachel Vincent, Lisa Vincent-Smith, Shico Visuvanathan, Alan Vuylsteke, Sam Waddy, Rachel Wake, Andrew Walden, Ingeborg Welters, Tony Whitehouse, Paul Whittaker, Ashley Whittington, Meme Wijesinghe, Martin Williams, Lawrence Wilson, Stephen Winchester, Martin Wiselka, Adam Wolverson, Daniel G. Wootton, Andrew Workman, Bryan Yates, Peter Young, Sarah E. McDonald, Victoria Shaw, Katie A. Ahmed, Jane A. Armstrong, Milton Ashworth, Innocent G. Asiimwe, Siddharth Bakshi, Samantha L. Barlow, Laura Booth, Benjamin Brennan, Katie Bullock, Nicola Carlucci, Emily Cass, Benjamin W. Catterall, Jordan J. Clark, Emily A. Clarke, Sarah Cole, Louise Cooper, Helen Cox, Christopher Davis, Oslem Dincarslan, Alejandra D. Carracedo, Chris Dunn, Philip Dyer, Angela Elliott, Anthony Evans, Lorna Finch, Lewis W. Fisher, Lisa Flaherty, Terry Foster, Isabel Garcia-Dorival, Philip Gunning, Catherine Hartley, Anthony Holmes, Rebecca L. Jensen, Christopher B. Jones, Trevor R. Jones, Shadia Khandaker, Katharine King, Robyn T. Kiy, Chrysa Koukorava, Annette Lake, Suzannah Lant, Diane Latawiec, Lara Lavelle-Langham, Daniella Lefteri, Lauren Lett, Lucia A. Livoti, Maria Mancini, Hannah Massey, Nicole Maziere, Sarah McDonald, Laurence McEvoy, John McLauchlan, Soeren Metelmann, Nahida S. Miah, Joanna Middleton, Joyce Mitchell, Ellen G. Murphy, Rebekah Penrice-Randal, Jack Pilgrim, Tessa Prince, Will Reynolds, P. M. Ridley, Debby Sales, Victoria E. Shaw, Rebecca K. Shears, Benjamin Small, Krishanthi S. Subramaniam, Agnieska Szemiel, Aislynn Taggart, Jolanta Tanianis-Hughes, Jordan Thomas, Erwan Trochu, Libby v. Tonder, Eve Wilcock, J. E. Zhang, Seán Keating, Cara Donegan, Rebecca G. Spencer, Chloe Donohue, Fiona Griffiths, Hayley Hardwick, Wilna Oosthuyzen, COVID-CNS Consortium, Adam Hampshire, Adam Sieradzki, Adam W. Seed, Afagh Garjani, Akshay Nair, Alaisdair Coles, Alan Carson, Alastair Darby, Alex Berry, Alex Dregan, Alexander Grundmann, Alish Palmos, Ammar Al-Chalabi, Andrew M. McIntosh, Angela E. Holland, Angela Roberts, Angela Vincent, Annalena Venneri, Anthony S. David, Arina Tamborska, Arvind Patel, Ava Easton, Benedict D. Michael, Bethan Blackledge, Bethany Facer, Bhagteshwar Singh, Brendan Sargent, Ceryce Collie, Charles Leek, Cherie Armour, Christopher M. Morris, Christopher M. Allen, Ciaran Mulholland, Claire L. MacIver, Cordelia Dunai, Craig J. Smith, Daniel J. van, Daniel Madarshahian, David Christmas, David Cousins, David K. Menon, David M. Christmas, David P. Breen, Dina Monssen, Edward Bullmore, Edward Needham, Emily McGlinchey, Emma Thomson, Eugene Duff, Eva M. Hodel, Ewan Harrison, Fernando Zelaya, Gabriella Lewis, Gavin McDonnell, Gerome Breen, Greta K. Wood, Guy B. Williams, C. Hannah, Henry C. Rogers, Ian Galea, Jacqueline Smith, Jade D. Harris, James B. Lilleker, Jay Amin, John P. Aggleton, John R. Bradley, John-Paul Taylor, Jonathan Cavanagh, Jonathan R. Coleman, Jonathan Underwood, Judith Breuer, Julian Hiscox, Karla Miller, Katherine C. Dodd, Kiran Glen, Laura Benjamin, Leonie Taams, Lily George, Marc Hardwick, Mark R. Baker, Masud Husain, Matthew Butler, Matthew Hotopf, Matthew R. Broome, Merna Samuel, Michael Griffiths, Michael P. Lunn, Michael S. Zandi, Monika Hartmann, Nadine Cossette, Naomi Martin, Nathalie Nicholas, Neil A. Harrison, Neil Basu, Neil Harrison, Nicholas Davies, Nicholas Wood, Nikos Evangelou, Obioma Orazulume, Pamela J. Shaw, Parisa Mansoori, Paul J. Harrison, Peter Jezzard, Peter M. Fernandes, Rachel Upthegrove, Rahul Batra, Rebecca Gregory, Rhys H. Thomas, Richard Bethlehem, Richard Francis, Ronan O’Malley, Rustam A. Salman, Ryan McIlwaine, Sandar Kyaw, Sarosh Irani, Savini Gunatilake, Scott Semple, Shahd H. Hamid, Sharon Peacock, Silvia Rota, Simon Keller, Sophie Pendered, Suzanne Barrett, Stella Hughes, Stella-Maria Paddick, Stephen J. Sawcer, Stephen Smith, Steven Williams, Sui H. Wong, Sylviane Defres, Thomas Jackson, Thomas M. Jenkins, Thomas Pollak, Timothy Nicholson, Tonny Veenith, Victoria Grimbly, Virginia Newcombe, Leonie S. Taams, David K. Menon

**Affiliations:** 1 Clinical Infection, Microbiology, and Immunology, Institute of Infection, Veterinary and Ecological Sciences, University of Liverpool, Liverpool, L69 7BE UK; 2 NIHR Health Protection Research Unit (HPRU) in Emerging and Zoonotic Infections at University of Liverpool, Liverpool, L69 7BE UK; 3 The Walton Centre NHS Foundation Trust, Liverpool, L9 7BB UK; 4 Department of Clinical Neurosciences, University of Cambridge, Cambridge, CB2 0QQ UK; 5 Division of Anaesthesia, Department of Medicine, University of Cambridge, Cambridge, CB2 0QQ UK; 6 Health Data Science, Institute of Population Health, University of Liverpool, Liverpool, L69 3GF UK; 7 Oxford Autoimmune Neurology Group, Nuffield Department of Clinical Neurosciences, University of Oxford, Oxford, OX3 9DU UK; 8 Departments of Neurology and Neuroscience, Mayo Clinic, Jacksonville, FL 32224 USA; 9 University of Liverpool, Liverpool, L69 7BE UK; 10 Department of Microbiology, Icahn School of Medicine, Mount Sinai, NY 10029 USA; 11 Center for Vaccine Research and Pandemic Preparedness (C-VARPP), Icahn School of Medicine, Mount Sinai, NY 10029 USA; 12 Infection Biology & Microbiomes, Institute of Infection, Veterinary and Ecological Sciences, University of Liverpool, Liverpool, L3 5RF UK; 13 Social, Genetic and Developmental Psychiatry Centre, Institute of Psychiatry, Psychology & Neuroscience, King’s College London, London, SE5 8AF UK; 14 NIHR Maudsley Biomedical Research Centre, King’s College London, London, SE5 8AF UK; 15 Centre for Cell Imaging, Liverpool Shared Research Facilities, Faculty of Health and Life Sciences, University of Liverpool, Liverpool, L69 7ZB UK; 16 NIHR BioResource, Cambridge University Hospitals NHS Foundation, Cambridge, CB2 0QQ UK; 17 Department of Haematology, University of Cambridge, Cambridge, CB2 0QQ UK; 18 Clinical Neurosciences, Clinical and Experimental Science, Faculty of Medicine, University of Southampton, Southampton, SO17 1BF UK; 19 Department of Neurology, Wessex Neurological Centre, University Hospital Southampton NHS Foundation Trust, Southampton, SO16 6YD UK; 20 Département de médecine interne des spécialités (DEMED), University of Geneva, Geneva, CH-1211 Switzerland; 21 The Pandemic Institute, Liverpool, L7 3FA UK; 22 University of Cambridge, Cambridge, CB2 0QQ UK; 23 Department of Medicine, School of Clinical Medicine, University of Cambridge, Cambridge, CB2 0QQ UK; 24 Centre for Immunology, School of Infection & Immunity, College of Medical, Veterinary & Life Sciences, University of Glasgow, Glasgow, G12 8TA UK; 25 Nuffield Department of Clinical Neurosciences, University of Oxford, Oxford, OX3 9DU UK; 26 Roslin Institute, University of Edinburgh, Edinburgh, EH25 9RG UK; 27 Intensive Care Unit, Royal Infirmary of Edinburgh, Edinburgh, EH10 5HF UK; 28 National Heart and Lung Institute, Imperial College London, London, SW7 2BX UK; 29 Imperial College Healthcare NHS Trust, London, W2 1NY UK; 30 Respiratory Unit, Alder Hey Children’s Hospital NHS Foundation Trust, Liverpool, L14 5AB UK; 31 Centre for Inflammation Biology and Cancer Immunology, King’s College London, London, SE1 9RT UK; 32 University of Edinburgh, Edinburgh, UK; 33 Imperial College London, London, UK; 34 University of Liverpool, Liverpool, UK; 35 Public Health England, London, UK; 36 MRC-University of Glasgow Centre for Virus Research, Glasgow, UK; 37 University of Sheffield, Sheffield, UK; 38 Liverpool School of Tropical Medicine, Liverpool, UK; 39 University of Birmingham, Birmingham, UK; 40 University College London, London, UK; 41 University of Oxford, Oxford, UK; 42 Nottingham University Hospitals NHS Trust, Nottingham, UK; 43 John Radcliffe Hospital, Oxford, UK; 44 King’s College London, London, UK; 45 University of Cambridge, Cambridge, UK; 46 Public Health Scotland, Edinburgh, UK; 47 ISARIC4C Investigators, Liverpool, UK; 48 University of Manchester, Manchester, UK; 49 King’s College Hospital, London, UK; 50 Royal Infirmary Edinburgh, Edinburgh, UK; 51 Roslin Institute, Edinburgh, UK; 52 COVID-CNS Consortium, York, UK; 53 Liverpool University Hospitals NHS Foundation Trust, Liverpool, UK; 54 University of Nottingham, Nottingham, UK; 55 Edinburgh University, Edinburgh, UK; 56 University of Southampton, Southampton, UK; 57 Nottingham University Hospital, Nottingham, UK; 58 University of Glasgow, Glasgow, UK; 59 Salford Royal Foundation Trust, Manchester, UK; 60 Queens University Belfast, Belfast, UK; 61 Newcastle University, Newcastle, UK; 62 Cardiff University, Cardiff, UK; 63 Sheffield Institute for Translational Neuroscience, Sheffield, UK; 64 Dundee University, Dundee, UK; 65 South London and Maudsley NHS Foundation Trust, London, UK; 66 Belfast Health and Social Care Trust, Belfast, UK; 67 COVID-CNS Consortium, Edinburgh, UK; 68 The University of Manchester, Manchester, UK; 69 Kings College London, London, UK; 70 Royal Infirmary of Edinburgh, Edinburgh, UK; 71 Aintree University Hospital, Liverpool, UK; 72 National Institute for Health Research (NIHR) Bioresource, London, UK; 73 Birmingham University, Birmingham, UK; 74 Sheffield Teaching Hospitals NHS Foundation Trust, Sheffield, UK; 75 Translational and Clinical Research, Newcastle, UK; 76 The Stroke Association, London, UK; 77 The University of Sheffield, Sheffield, UK; 78 The University of Edinburgh, Edinburgh, UK; 79 Institute of Mental health, Nottingham, UK; 80 Royal Stoke University Hospital, Stoke on Trent, UK; 81 Northern Health and Social Care Trust, Belfast, UK

**Keywords:** Neurological manifestations, Neurological disorders, Inflammation, SARS-CoV-2

Correction to: *Nature Communications* 10.1038/s41467-023-42320-4, published online 22 December 2023

The original version of this article omitted three members of the COVID-CNS Consortium. Alex Berry and Obioma Orazulume, who are from the ‘University College London, London, UK’, and Ian Galea, who is from the ‘University of Southampton, Southampton, UK’ were added to the list of COVID-CNS Consortium members. This has been corrected in both the PDF and HTML versions of the article.

