# Supplementary material for: Para-infectious brain injury in COVID-19 persists at follow-up despite attenuated cytokine and autoantibody responses
Source: Nat Commun. 2023 Dec 22;14:8487. doi: 10.1038/s41467-023-42320-4 (PMC10746705; doi:10.1038/s41467-023-42320-4)
Supplement: Supplementary file 1 — Supplementary Information [file 41467_2023_42320_MOESM1_ESM.pdf]

Supplementary Table 1: Median values of biomarkers

| | Brain Injury Markers | | | | Cytokines | | | | | |
|---|---|---|---|---|---|---|---|---|---|---|
| **Median (pg/mL) and (Q1, Q3)** | **NfL** | **GFAP** | **Tau** | **UCH-L1** | **IL-1RA** | **IL-6** | **IL-12p40** | **CCL2** | **M-CSF** | **HGF** |
| **ISARIC GCS=15 (n=76)** | 26.5 (12.9, 62.3) | 125 (86.9, 230.0) | 1.1 (0.9, 1.4) | 21.9 (11.3, 36.3) | 772.5 (534.8, 1722.3) | 2.12 (1.4, 12.8) | 166.5 (109.3, 253.8) | 53.3 (36.6, 69.6) | 46.6 (28.5, 67.1) | 809.1 (530.5, 1505.0) |
| **ISARIC GCS≤14 (n=35)** | 58.2 (30.7, 122.8) | 151 (90.5, 263.0) | 1.2 (0.9, 1.7) | 33.7 (18.8, 48.5) | 1501.3 (1093.0, 3128.8) | 33.8 (12.6, 72.6) | 286.1 (224.1, 395.2) | 132.8 (55.5, 208.4) | 75.9 (54.0, 107.1) | 1965.0 (1570.0, 2956.6) |
| **COVID-CNS COVID (n=36)** | 12.6 (8.3, 27.9) | 92.9 (61.3, 197.0) | 1.3 (1.1, 1.9) | 7.4 (4.0, 19.6) | 290.0 (197.2, 476.0) | 1.4 (1.4, 5.8) | 123.7 (68.4, 268.9) | 32.10 (23.1, 49.2) | 29.1 (17.6, 51.6) | 668.0 (385.9, 1693.2) |
| **COVID-CNS Neuro-COVID (n=56)** | 32.3 (17.9, 111.8) | 106.5 (79.5, 191.0) | 1.7 (1.3, 2.2) | 9.3 (5.3, 20.9) | 303.1 (218.1, 546.7) | 1.36 (1.36, 1.36) | 108.8 (91.1, 141.9) | 32.5 (20.0, 50.0) | 24.4 (16.0, 30.5) | 529.9 (405.2, 727.6) |

# Supplementary Table 2: COVID-CNS Neuro-COVID cases

| | Condition, n (%) | Diagnosis | n |
|---|---|---|---|
| **Central Nervous System Condition 41 (73%)** | Cerebrovascular 12 (21%) | Ischaemic stroke | 6 |
| | | Haemorrhagic stroke | 2 |
| | | Cerebral venous sinus thrombosis | 1 |
| | | Vasculitic stroke | 1 |
| | | CNS vasculitis | 1 |
| | | PRES* | 1 |
| | CNS inflammation 9 (16%) | Encephalitis | 4 |
| | | Demyelinating** | 5 |
| | Movement disorder 3 (5.4%) | Hyperkinetic movement disorder | 3 |
| | Seizures and others 17 (30%) | Seizures | 8 |
| | | Encephalopathy | 5 |
| | | Neuropsychiatric | 2 |
| | | Others | 2 |
| **Peripheral Nervous System Condition 15 (27%)** | Various PNS 15 (27%) | Peripheral nerves dysfunction | 11 |
| | | Myopathy | 2 |
| | | Cranial nerve palsy | 2 |

*PRES = posterior reversible encephalopathy syndrome
**Includes: multiple sclerosis, Acute disseminated encephalomyelitis (ADEM), Chronic relapsing inflammatory optic neuritis (CRION)

# Supplementary Table 3: ISARIC cohort cytokines correlations with brain injury markers (Pearson's coefficients)

| | Variables | NFL | | GFAP | | Tau | | UCHL | |
|---|---|---|---|---|---|---|---|---|---|
| | | Correlation | Adj p-value (Estimate) | Correlation | Adj p-value (Estimate) | Correlation | Adj p-value (Estimate) | Correlation | Adj p-value (Estimate) |
| 1 | **CCL5** | -0.168 | 1.000 | -0.190 | 1.000 | -0.157 | 1.000 | -0.023 | 1.000 |
| 2 | **SCGF-beta** | -0.086 | 1.000 | 0.018 | 1.000 | -0.020 | 1.000 | -0.012 | 1.000 |
| 3 | **CXCL10** | -0.056 | 1.000 | -0.086 | 1.000 | -0.031 | 1.000 | -0.042 | 1.000 |
| 4 | **IL-6** | -0.013 | 1.000 | -0.054 | 1.000 | -0.007 | 1.000 | 0.081 | 1.000 |
| 5 | **CCL2** | 0.118 | 1.000 | 0.106 | 1.000 | 0.098 | 1.000 | 0.070 | 1.000 |
| 6 | **CCL27** | 0.128 | 1.000 | 0.124 | 1.000 | 0.125 | 1.000 | 0.054 | 1.000 |
| 7 | **Eotaxin** | 0.091 | 1.000 | 0.056 | 1.000 | 0.161 | 1.000 | -0.019 | 1.000 |
| 8 | **CXCL9** | 0.120 | 1.000 | -0.067 | 1.000 | 0.075 | 1.000 | -0.004 | 1.000 |
| 9 | **IL-2Ra** | 0.067 | 1.000 | 0.019 | 1.000 | 0.064 | 1.000 | 0.114 | 1.000 |
| 10 | **HGF** | 0.616 | <0.001* (0.033) | 0.250 | 0.773 | 0.682 | <0.001* (0.0005) | 0.156 | 1.000 |
| 11 | **IL-12(p40)** | 0.632 | <0.001* (0.414) | 0.401 | 0.002* (1.027) | 0.651 | <0.001* (0.005) | 0.367 | 0.004* (0.096) |
| 12 | **IL-10** | 0.180 | 1.000 | 0.399 | 0.004* (30.74) | 0.119 | 1.000 | 0.610 | <0.001* (4.932) |
| 13 | **TNF** | 0.239 | 0.464 | 0.460 | <0.001* (9.518) | 0.167 | 1.000 | 0.561 | <0.001* (1.246) |
| 14 | **IL-2** | 0.222 | 1.000 | 0.534 | <0.001* (42.03) | 0.112 | 1.000 | 0.764 | <0.001* (6.439) |
| 15 | **IL-3** | 0.212 | 1.000 | 0.567 | <0.001* (965.6) | 0.140 | 1.000 | 0.758 | <0.001* (137.331) |
| 16 | **LTalpha** | -0.077 | 1.000 | 0.204 | 1.000 | -0.078 | 1.000 | 0.386 | 0.002* (0.184) |
| 17 | **IL-9** | 0.003 | 1.000 | 0.266 | 0.288 | 0.030 | 1.000 | 0.487 | <0.001* (0.307) |
| 18 | **IL-12(p70)** | 0.093 | 1.000 | 0.330 | 0.091 | 0.095 | 1.000 | 0.379 | 0.004* (1.807) |
| 19 | **IL-13** | -0.040 | 1.000 | 0.047 | 1.000 | 0.013 | 1.000 | 0.052 | 1.000 |
| 20 | **GM-CSF** | 0.060 | 1.000 | 0.110 | 1.000 | 0.046 | 1.000 | 0.154 | 1.000 |
| 21 | **IL-5** | 0.065 | 1.000 | 0.154 | 1.000 | 0.045 | 1.000 | 0.299 | 0.116 |
| 22 | **b-NGF** | 0.041 | 1.000 | 0.180 | 1.000 | 0.007 | 1.000 | 0.407 | 0.001* (5.598) |
| 23 | **CCL4** | -0.009 | 1.000 | -0.007 | 1.000 | -0.051 | 1.000 | 0.347 | 0.015* (0.115) |
| 24 | **IL-15** | 0.003 | 1.000 | -0.035 | 1.000 | -0.050 | 1.000 | 0.273 | 0.258 |
| 25 | **IL-8** | 0.010 | 1.000 | -0.025 | 1.000 | -0.028 | 1.000 | 0.297 | 0.109 |
| 26 | **CCL3** | -0.003 | 1.000 | -0.034 | 1.000 | -0.043 | 1.000 | 0.282 | 0.193 |
| 27 | **G-CSF** | -0.023 | 1.000 | -0.019 | 1.000 | -0.030 | 1.000 | 0.296 | 0.120 |
| 28 | **VEGF** | 0.013 | 1.000 | -0.055 | 1.000 | -0.077 | 1.000 | 0.187 | 1.000 |
| 29 | **CXCL1** | -0.049 | 1.000 | 0.006 | 1.000 | -0.091 | 1.000 | 0.205 | 1.000 |
| 30 | **TRAIL** | 0.028 | 1.000 | 0.264 | 0.498 | 0.046 | 1.000 | 0.441 | <0.001* (4.545) |
| 31 | **IL-1alpha** | 0.227 | 1.000 | 0.306 | 0.299 | 0.144 | 1.000 | 0.528 | <0.001* (1.717) |
| 32 | **FGF basic** | 0.305 | 0.118 | 0.278 | 0.468 | 0.279 | 0.241 | 0.408 | 0.001* (1.032) |
| 33 | **IFN-alpha2** | 0.173 | 1.000 | 0.006 | 1.000 | 0.178 | 1.000 | 0.296 | 0.110 |
| 34 | **IL-4** | 0.191 | 1.000 | 0.119 | 1.000 | 0.195 | 1.000 | 0.248 | 0.673 |
| 35 | **IL-17A** | 0.244 | 1.000 | 0.150 | 1.000 | 0.242 | 1.000 | 0.303 | 0.109 |
| 36 | **LIF** | 0.119 | 1.000 | 0.044 | 1.000 | 0.073 | 1.000 | 0.273 | 0.316 |
| 37 | **MIF** | 0.037 | 1.000 | -0.056 | 1.000 | -0.008 | 1.000 | 0.191 | 1.000 |
| 38 | **IL-1Ra** | 0.188 | 1.000 | 0.017 | 1.000 | 0.079 | 1.000 | 0.247 | 0.572 |
| 39 | **CCL7** | 0.098 | 1.000 | 0.045 | 1.000 | 0.022 | 1.000 | 0.140 | 1.000 |
| 40 | **IFN-gamma** | 0.194 | 1.000 | 0.169 | 1.000 | 0.163 | 1.000 | 0.276 | 0.157 |
| 41 | **SCF** | 0.334 | 0.007* (0.419) | 0.113 | 0.889 | 0.304 | 0.009* (0.005) | 0.237 | 0.213 |
| 42 | **M-CSF** | 0.293 | 0.094 | 0.112 | 1.000 | 0.211 | 0.727 | 0.234 | 0.359 |
| 43 | **IL-16** | 0.206 | 1.000 | 0.117 | 1.000 | 0.156 | 1.000 | 0.148 | 1.000 |
| 44 | **IL-18** | 0.205 | 1.000 | 0.036 | 1.000 | 0.113 | 1.000 | 0.173 | 1.000 |
| 45 | **IL-1beta** | 0.195 | 1.000 | 0.112 | 1.000 | 0.160 | 1.000 | 0.328 | 0.037* (7.381) |
| 46 | **PDGF-bb** | -0.065 | 1.000 | -0.125 | 1.000 | -0.163 | 1.000 | 0.114 | 1.000 |
| 47 | **CXCL12** | 0.081 | 1.000 | 0.140 | 1.000 | 0.118 | 1.000 | 0.271 | 0.375 |
| 48 | **IL-7** | -0.001 | 1.000 | 0.024 | 1.000 | -0.030 | 1.000 | 0.121 | 1.000 |

# Supplementary Table 4: ISARIC Median-centred data
# Differences between GCS=15 and GCS≤14
# (two-tailed Mann-Whitney tests)

| Count | Cytokines | Adj p-value[1]<br>(test,p-value[2]) | Effect size |
|---|---|---|---|
| 1 | CCL5 | 1<br>(M, <0.001) | -0.018 |
| 2 | SCGF-beta | 1<br>(M, <0.001) | -0.042 |
| 3 | CXCL10 | 0.717<br>(M, <0.001) | -0.232 |
| 4 | IL-6 | <0.001*<br>(M, <0.001) | -0.429 |
| 5 | CCL2 | 0.137<br>(M, <0.001) | -0.284 |
| 6 | CCL27 | 0.16<br>(M, <0.001) | 0.279 |
| 7 | Eotaxin | <0.001*<br>(M, <0.001) | 0.436 |
| 8 | CXCL9 | 1<br>(M, <0.001) | -0.103 |
| 9 | IL-2Ra | 1<br>(M, <0.001) | -0.028 |
| 10 | HGF | <0.001*<br>(M, <0.001) | -0.425 |
| 11 | IL-12(p40) | 0.034*<br>(M, <0.001) | -0.322 |
| 12 | IL-10 | 1<br>(M, <0.001) | -0.156 |
| 13 | TNF | 1<br>(M, <0.001) | 0.04 |
| 14 | IL-2 | 1<br>(M, <0.001) | 0.116 |
| 15 | IL-3 | 0.022*<br>(M, <0.001) | 0.333 |
| 16 | LTalpha | 0.168<br>(M, <0.001) | 0.277 |
| 17 | IL-9 | 0.003*<br>(M, <0.001) | 0.383 |
| 18 | IL-12(p70) | 0.127<br>(M, <0.001) | 0.286 |
| 19 | IL-13 | 1<br>(M, <0.001) | 0.07 |
| 20 | GM-CSF | 1<br>(M, <0.001) | -0.02 |
| 21 | IL-5 | 0.019*<br>(M, <0.001) | 0.336 |
| 22 | b-NGF | 1<br>(M, <0.001) | 0.216 |
| 23 | CCL4 | 0.002*<br>(M, <0.001) | 0.388 |
| 24 | IL-15 | 0.125<br>(M, <0.001) | 0.286 |
| 25 | IL-8 | 0.453<br>(M, <0.001) | -0.247 |
| 26 | CCL3 | 1<br>(M, <0.001) | -0.103 |
| 27 | G-CSF | 1<br>(M, <0.001) | -0.011 |
| 28 | VEGF | 1<br>(M, <0.001) | 0.033 |
| 29 | CXCL1 | 1<br>(M, <0.001) | -0.066 |
| 30 | TRAIL | 0.014*<br>(M, <0.001) | 0.345 |
| 31 | IL-1alpha | 1<br>(M, <0.001) | 0.012 |
| 32 | FGF basic | 0.018*<br>(M, <0.001) | 0.339 |
| 33 | IFN-alpha2 | 1<br>(M, <0.001) | -0.093 |
| 34 | IL-4 | <0.001*<br>(M, <0.001) | 0.46 |
| 35 | IL-17A | 1<br>(M, <0.001) | -0.022 |
| 36 | LIF | 1<br>(M, <0.001) | 0.154 |
| 37 | MIF | 1<br>(M, <0.001) | -0.066 |
| 38 | IL-1Ra | 0.071*<br>(M, <0.001) | -0.302 |
| 39 | CCL7 | 1<br>(M, <0.001) | -0.092 |
| 40 | IFN-gamma | 1<br>(M, <0.001) | -0.135 |
| 41 | SCF | 1<br>(M, <0.001) | 0.185 |
| 42 | M-CSF | 0.611<br>(M, <0.001) | -0.236 |
| 43 | IL-16 | 1<br>(M, <0.001) | -0.145 |
| 44 | IL-18 | 1<br>(M, <0.001) | -0.204 |
| 45 | IL-1beta | 0.421<br>(M, <0.001) | 0.249 |
| 46 | PDGF-bb | 1<br>(M, <0.001) | -0.146 |
| 47 | CXCL12 | 1<br>(M, <0.001) | 0.102<br>(M, <0.001) |
| 48 | IL-7 | 1<br>(M, <0.001) | 0.001<br>(M, <0.001) |

Supplementary Table 5: Correlations of cytokines and brain injury markers in the COVID-CNS Neuro-COVID cohort with regression models (Pearson's coeffecients)

| Variables | NFL | | GFAP | | Tau | | UCHL | |
|---|---|---|---|---|---|---|---|---|
| | Correlation | Adj p-value | Correlation | Adj p-value | Correlation | Adj p-value | Correlation | Adj p-value |
| **CCL5** | -0.245 | 1.000 | -0.222 | 1.000 | 0.158 | 1.000 | 0.128 | 1.000 |
| **SCGF-beta** | 0.169 | 1.000 | 0.283 | 1.000 | 0.256 | 1.000 | 0.100 | 1.000 |
| **CXCL10** | 0.096 | 1.000 | 0.078 | 1.000 | 0.416 | 0.140 | 0.069 | 1.000 |
| **IL-6** | -0.059 | 1.000 | -0.010 | 1.000 | 0.003 | 1.000 | -0.043 | 1.000 |
| **CCL2** | 0.083 | 1.000 | 0.128 | 1.000 | 0.492 | 0.016* | 0.022 | 1.000 |
| **CCL27** | 0.183 | 1.000 | 0.032 | 1.000 | 0.255 | 1.000 | 0.006 | 1.000 |
| **Eotaxin** | -0.042 | 1.000 | 0.010 | 1.000 | -0.077 | 1.000 | -0.100 | 1.000 |
| **CXCL9** | 0.017 | 1.000 | -0.059 | 1.000 | 0.132 | 1.000 | 0.159 | 1.000 |
| **IL-2Ra** | 0.104 | 1.000 | 0.049 | 1.000 | 0.547 | 0.002* | 0.517 | 0.007* |
| **HGF** | 0.251 | 1.000 | 0.162 | 1.000 | 0.238 | 1.000 | 0.084 | 1.000 |
| **IL-12(p40)** | 0.298 | 1.000 | 0.116 | 1.000 | 0.200 | 1.000 | 0.124 | 1.000 |
| **IL-10** | -0.068 | 1.000 | -0.004 | 1.000 | 0.151 | 1.000 | -0.032 | 1.000 |
| **TNF** | 0.064 | 1.000 | 0.167 | 1.000 | 0.409 | 0.165 | 0.042 | 1.000 |
| **IL-2** | 0.064 | 1.000 | 0.111 | 1.000 | 0.123 | 1.000 | -0.129 | 1.000 |
| **IL-3** | NA | NA | NA | NA | NA | NA | NA | NA |
| **LTalpha** | 0.091 | 1.000 | 0.161 | 1.000 | -0.069 | 1.000 | -0.071 | 1.000 |
| **IL-9** | -0.009 | 1.000 | 0.075 | 1.000 | -0.051 | 1.000 | -0.082 | 1.000 |
| **IL-12(p70)** | 0.043 | 1.000 | -0.123 | 1.000 | -0.139 | 1.000 | -0.028 | 1.000 |
| **IL-13** | -0.008 | 1.000 | -0.079 | 1.000 | -0.159 | 1.000 | -0.049 | 1.000 |
| **GM-CSF** | -0.079 | 1.000 | -0.091 | 1.000 | -0.161 | 1.000 | -0.038 | 1.000 |
| **IL-5** | -0.068 | 1.000 | -0.063 | 1.000 | -0.053 | 1.000 | -0.054 | 1.000 |
| **b-NGF** | -0.078 | 1.000 | -0.070 | 1.000 | -0.044 | 1.000 | -0.042 | 1.000 |
| **CCL4** | 0.143 | 1.000 | 0.176 | 1.000 | -0.044 | 1.000 | -0.036 | 1.000 |
| **IL-15** | -0.031 | 1.000 | -0.016 | 1.000 | -0.080 | 1.000 | -0.009 | 1.000 |
| **IL-8** | -0.172 | 1.000 | -0.118 | 1.000 | 0.246 | 1.000 | 0.152 | 1.000 |
| **CCL3** | 0.094 | 1.000 | 0.052 | 1.000 | 0.006 | 1.000 | 0.008 | 1.000 |
| **G-CSF** | 0.086 | 1.000 | 0.044 | 1.000 | 0.031 | 1.000 | 0.001 | 1.000 |
| **VEGF** | -0.078 | 1.000 | -0.071 | 1.000 | -0.009 | 1.000 | -0.039 | 1.000 |
| **CXCL1** | -0.060 | 1.000 | -0.067 | 1.000 | -0.171 | 1.000 | -0.050 | 1.000 |
| **TRAIL** | 0.009 | 1.000 | -0.022 | 1.000 | 0.167 | 1.000 | -0.080 | 1.000 |
| **IL-1alpha** | -0.006 | 1.000 | -0.012 | 1.000 | 0.069 | 1.000 | 0.043 | 1.000 |
| **FGF basic** | 0.259 | 1.000 | 0.189 | 1.000 | 0.251 | 1.000 | 0.141 | 1.000 |
| **IFN-alpha2** | 0.034 | 1.000 | 0.052 | 1.000 | 0.368 | 0.432 | -0.003 | 1.000 |
| **IL-4** | -0.006 | 1.000 | 0.034 | 1.000 | -0.009 | 1.000 | -0.015 | 1.000 |
| **IL-17A** | -0.074 | 1.000 | 0.004 | 1.000 | 0.002 | 1.000 | -0.108 | 1.000 |
| **LIF** | -0.100 | 1.000 | -0.087 | 1.000 | 0.103 | 1.000 | 0.135 | 1.000 |
| **MIF** | 0.061 | 1.000 | 0.102 | 1.000 | 0.303 | 1.000 | 0.074 | 1.000 |
| **IL-1Ra** | 0.069 | 1.000 | 0.185 | 1.000 | 0.582 | 0.001* | 0.039 | 1.000 |
| **CCL7** | 0.033 | 1.000 | 0.158 | 1.000 | 0.608 | <0.001* | 0.050 | 1.000 |
| **IFN-gamma** | -0.035 | 1.000 | 0.019 | 1.000 | 0.177 | 1.000 | 0.003 | 1.000 |
| **SCF** | 0.039 | 1.000 | 0.119 | 1.000 | 0.621 | <0.001* | 0.084 | 1.000 |
| **M-CSF** | 0.077 | 1.000 | 0.139 | 1.000 | 0.680 | <0.001* | 0.231 | 1.000 |
| **IL-16** | 0.048 | 1.000 | 0.157 | 1.000 | 0.578 | 0.001* | 0.098 | 1.000 |
| **IL-18** | 0.132 | 1.000 | 0.190 | 1.000 | 0.724 | <0.001* | 0.317 | 1.000 |
| **IL-1beta** | 0.100 | 1.000 | -0.007 | 1.000 | 0.337 | 0.844 | 0.072 | 1.000 |
| **PDGF-bb** | 0.181 | 1.000 | 0.186 | 1.000 | -0.005 | 1.000 | -0.058 | 1.000 |
| **CXCL12** | 0.107 | 1.000 | 0.201 | 1.000 | 0.091 | 1.000 | -0.091 | 1.000 |
| **IL-7** | -0.030 | 1.000 | -0.007 | 1.000 | -0.029 | 1.000 | -0.040 | 1.000 |

# Supplementary Table 6: List of antigens on protein microarray

| Number | Antigen | Type | Number | Antigen | Type | Number | Antigen | Type |
|---|---|---|---|---|---|---|---|---|
| 1 | ACE | Ubiquitous | 52 | GNRHR | Endocrine | 103 | MYL4 | Cardiac |
| 2 | ACTA1 | Muscle | 53 | GRIA2 | Central Nervous System | 104 | MYL7 | Cardiac |
| 3 | ADAMTS13 | Coagulation | 54 | GRIA3 | Central Nervous System | 105 | NEFL | Central Nervous System |
| 4 | AGER | Lung | 55 | GRIA4 | Central Nervous System | 106 | NOVA1 | Central Nervous System |
| 5 | AGTR1 | Kidney | 56 | GRIN1 | Central Nervous System | 107 | NPHS2 | Kidney |
| 6 | ANKRD23 | Muscle | 57 | GRIN2A | Central Nervous System | 108 | NPPA | Cardiac |
| 7 | ANXA4 | Central Nervous System | 58 | GRIN3A | Central Nervous System | 109 | NPPB | Cardiac |
| 8 | ANXA5 | Coagulation | 59 | GRIN3B | Central Nervous System | 110 | Nucleocapsid protein | SARS-CoV-2 |
| 9 | APOH | Coagulation | 60 | GRINA | Central Nervous System | 111 | NUP210 | Ubiquitous |
| 10 | APP | Central Nervous System | 61 | GRM1 | Central Nervous System | 112 | OMG | Central Nervous System |
| 11 | BSG | Central Nervous System | 62 | GRM2 | Central Nervous System | 113 | PLA2R1 | Kidney |
| 12 | CD74 | HLA | 63 | GRM3 | Central Nervous System | 114 | PNMA1 | Central Nervous System |
| 13 | CDH1 | Ubiquitous | 64 | GRM4 | Central Nervous System | 115 | PNMA2 | Central Nervous System |
| 14 | CDH13 | Central Nervous System | 65 | GRM7 | Central Nervous System | 116 | POMC | Endocrine |
| 15 | CDR2 | Central Nervous System | 66 | GRM8 | Central Nervous System | 117 | PPP1R27 | Muscle |
| 16 | CEACAM1 | Ubiquitous | 67 | GSTT1 | Kidney | 118 | PRL | Endocrine |
| 17 | CEACAM5 | Ubiquitous | 68 | HARS | Muscle | 119 | PROC | Coagulation |
| 18 | CENPB | Ubiquitous | 69 | HLA-A | HLA | 120 | PROS1 | Coagulation |
| 19 | CENPH | Ubiquitous | 70 | HLA-B | HLA | 121 | PRTN3 | Kidney |
| 20 | CHRM2 | Cardiac | 71 | HLA-C | HLA | 122 | S100B | Central Nervous System |
| 21 | CHRNA10 | Muscle | 72 | HLA-DMA | HLA | 123 | SCGB1A1 | Lung |
| 22 | CHRNA9 | Central Nervous System | 73 | HLA-DMB | HLA | 124 | SCGB3A2 | Lung |
| 23 | CLDN5 | BBB | 74 | HLA-DOA | HLA | 125 | SELE | BBB |
| 24 | COL1A1 | Lung | 75 | HLA-DOB | HLA | 126 | SFTPA1 | Lung |
| 25 | COL1A2 | Lung | 76 | HLA-DPA1 | HLA | 127 | SFTPA2 | Lung |
| 26 | COL4a3 | Kidney | 77 | HLA-DPB1 | HLA | 128 | SFTPC | Lung |
| 27 | COL4A3BP | Central Nervous System | 78 | HLA-DQA1 | HLA | 129 | SLC22A12 | Kidney |
| 28 | COL5A2 | Lung | 79 | HLA-DQB1 | HLA | 130 | SLC2A1 | BBB |
| 29 | DBT | Ubiquitous | 80 | HLA-DQB2 | HLA | 131 | SNCA | Central Nervous System |
| 30 | DCN | Central Nervous System | 81 | HLA-DRA | HLA | 132 | Spike protein | SARS-CoV-2 |
| 31 | DDC | Ubiquitous | 82 | HLA-DRB1 | HLA | 133 | SSB | Central Nervous System |
| 32 | DLAT | Ubiquitous | 83 | HLA-DRB3 | HLA | 134 | TGM2 | Ubiquitous |
| 33 | DPYSL5 | Central Nervous System | 84 | HLA-DRB4 | HLA | 135 | TJP1 | BBB |
| 34 | DRD2 | Central Nervous System | 85 | HLA-DRB5 | HLA | 136 | TMEM174 | Kidney |
| 35 | DUPD1 | Muscle | 86 | HLA-E | HLA | 137 | TNNI3 | Cardiac |
| 36 | EDNRA | Cardiac | 87 | HLA-F | HLA | 138 | TNNT2 | Cardiac |
| 37 | ELAVL4 | Central Nervous System | 88 | HLA-G | HLA | 139 | TPH1 | Central Nervous System |
| 38 | F2 | Coagulation | 89 | IDI2 | Muscle | 140 | TPM1 | Muscle |
| 39 | F7 | Coagulation | 90 | IFNA1 | Ubiquitous | 141 | TPO | Endocrine |
| 40 | F8 | Coagulation | 91 | IGHG1 | Immune system | 142 | TROVE2 | Central Nervous System |
| 41 | F9 | Coagulation | 92 | KCNJ10 | Central Nervous System | 143 | TSHB | Endocrine |
| 42 | FGB | Coagulation | 93 | KRT18 | Ubiquitous | 144 | TSHR | Endocrine |
| 43 | FSHB | Endocrine | 94 | LAMC2 | BBB | 145 | TTN | Muscle |
| 44 | GABBR1 | Central Nervous System | 95 | LGI1 | Central Nervous System | 146 | TUBA1B | Lung |
| 45 | GABRA1 | Central Nervous System | 96 | LRRC10 | Cardiac | 147 | TUBB3 | Central Nervous System |
| 46 | GABRB3 | Central Nervous System | 97 | MAG | Central Nervous System | 148 | UCHL1 | Central Nervous System |
| 47 | GAD1 | Central Nervous System | 98 | MAPT | Central Nervous System | 149 | UCP3 | Muscle |
| 48 | GAD2 | Central Nervous System | 99 | MBP | Central Nervous System | 150 | UMOD | Kidney |
| 49 | GFAP | Central Nervous System | 100 | MOG | Central Nervous System | 151 | VIM | Kidney |
| 50 | GHRHR | Endocrine | 101 | MPO | Kidney | 152 | ZIC4 | Central Nervous System |
| 51 | GLRA1 | Central Nervous System | 102 | MYBPHL | Cardiac | 153 | ZNF397 | Ubiquitous |

# Supplementary Table 7: COVID-CNS cohort co-morbidities

| Condition | COVID | Neuro-COVID |
|---|---|---|
| Frailty Scale Score (Median, Q1, Q3) | 2.5 (2,4) | 2.0 (2,3) |
| Hypertension | 36% | 29% |
| Tobacco smoking | 8% | 2% |
| eCigarette smoking | 0% | 0% |
| Hypercholesterolaemia | 6% | 9% |
| Muscular disease | 0% | 0% |
| Multiple sclerosis | 0% | 2% |
| Movement disorder | 0% | 0% |
| Dementia | 0% | 0% |
| Epilepsy | 6% | 4% |
| Ischemic stroke | 0% | 0% |
| Hemorrhagic stroke | 3% | 0% |
| Diabetes | 19% | 21% |
| Motor neuron disease | 0% | 0% |
| Neurological disease | 3% | 0% |
| Atrial fibrillation | 3% | 2% |
| HIV | 3% | 0% |
| Psychiatric/psychological disorder | 11% | 25% |
| Brain cancer | 0% | 0% |
| Cardiac disease | 3% | 4% |
| Chronic kidney disease | 14% | 4% |
| TB | 0% | 0% |
| Malignant neoplasm | 8% | 4% |
| Myasthenia gravis | 0% | 0% |
| Developmental delay | 0% | 0% |
| Chronic pulmonary disease | 17% | 11% |
| Chronic liver disease | 3% | 2% |
| Autoimmune disease | 3% | 7% |
| Known treatment with anti-epileptic or sedative | 3% | 4% |
| Known treatment with immunomodulation | 56%* | 61%** |

*COVID– corticosteroid administration
One additional participant with with tacrolimus

**Neuro-COVID– corticosteroid administration
One with tocilizumab, one additional participant with baricitinib and dimethyl fumarate

**a**

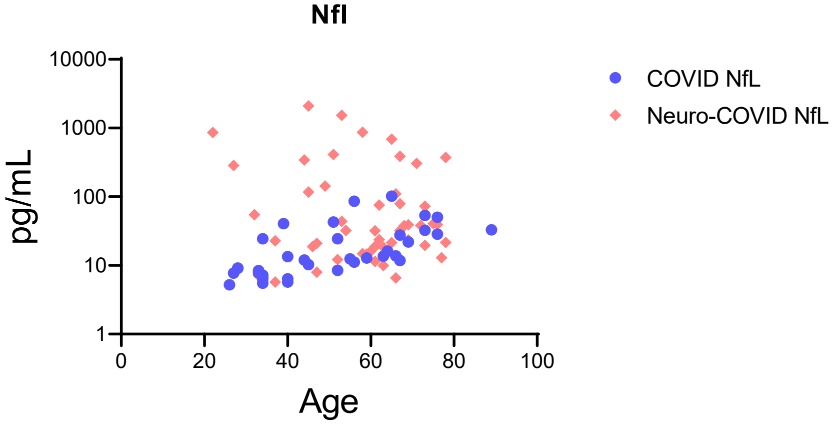


**b**

| Multiple regression model with age as a co-variate | | |
|---|---|---|
| Biomarker | Neuro-COVID/COVID estimate | p-value |
| NfL | 186.3 | 0.007 |
| GFAP | 56.0 | 0.512 |
| Tau | 0.6 | 0.070 |
| UCHL1 | 12.7 | 0.250 |

**c**

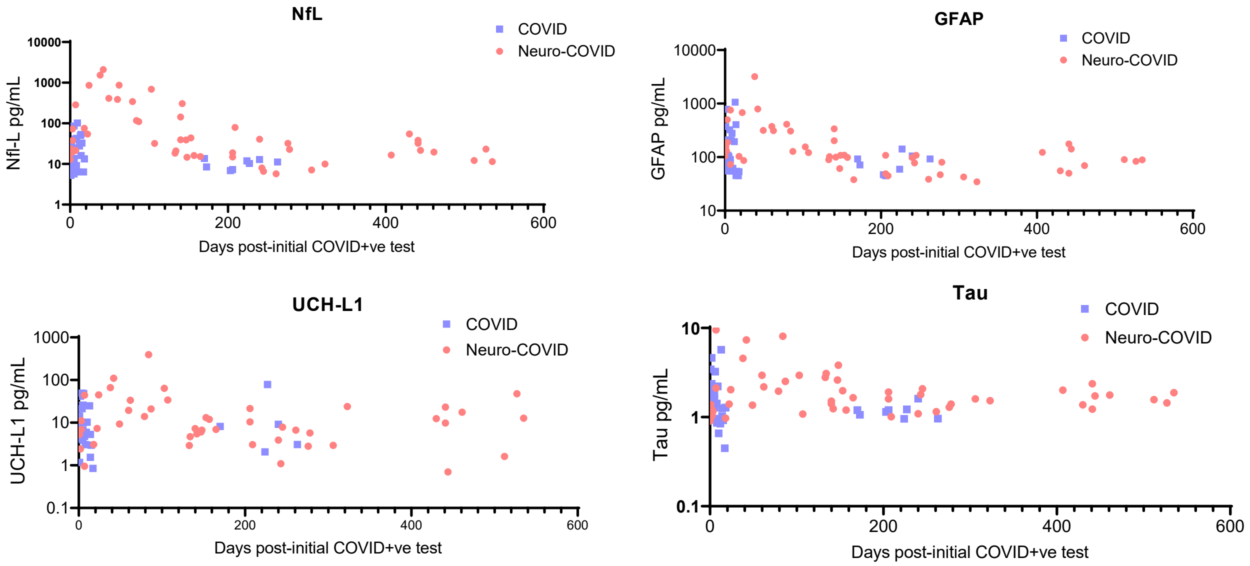


**Supplementary Fig. 1: COVID-CNS Brain injury markers stratified by age and over time**

(a) Serum NfL was measured by Simoa across ages of the participants in COVID control vs Neuro-COVID cases groups. The results show that age is not a major determinant of the overall difference. *Pearson's correlation p<0.05 in the COVID control group only

(b) Multiple regression model with age as a co-variate p-values for the four brain injury markers (NfL, GFAP, UCH-L1 and tTau)

(c) NfL, GFAP, UCH-L1 and Tau were measured by Simoa and plotted over time since initial COVID+ve test in the COVID-CNS (early and late convalescent participants)

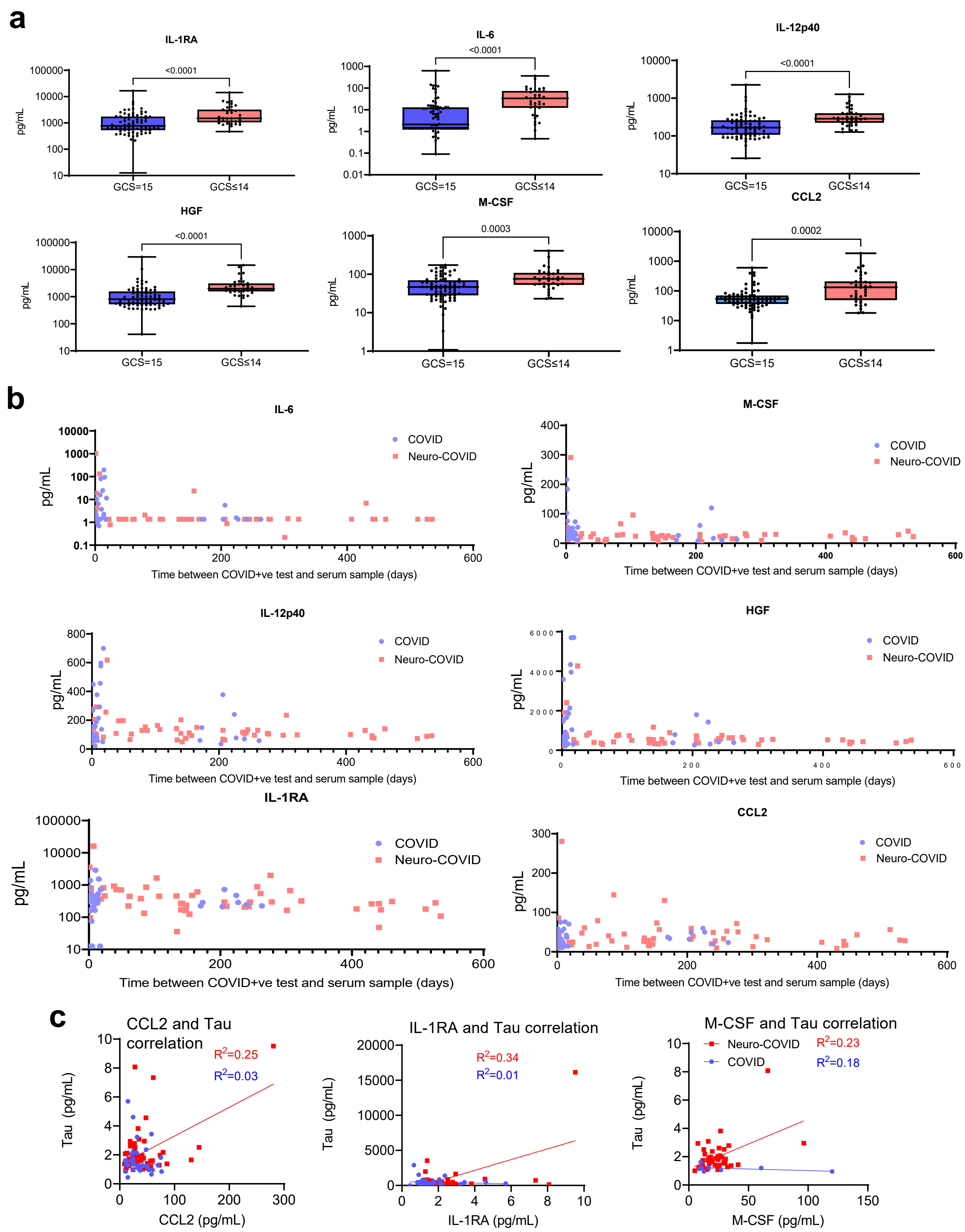


**Supplementary Fig. 2: Cytokine comparisons**

(a) Six cytokines measured by luminex were significantly higher in the sera of ISARIC participants (acute timepoints) with GCS≤14 compared to GCS=15 group. Two-tailed Mann-Whitney tests.

(b) The six serum cytokines plotted over time since initial COVID+ve test in the COVID-CNS (sub-acute/convalescent) cohort

(c) Significant correlations of three cytokines of interest with Tau in the COVID-CNS Neuro-COVID cohort (sub-acute/convalescent) with Pearson’s correlations shown

**a**

ISARIC: GCS≤14/GCS=15

| Protein | p_value | log2FC |
|---|---|---|
| IL-8 | 0.0016 | -2.097 |
| CCL3 | 0.0066 | -1.424 |
| IL-16 | 0.0072 | 0.610 |
| IL-2Ralpha | 0.0221 | 0.647 |
| IL-12p40 | 0.0000 | 0.673 |
| IFNgamma | 0.0042 | 0.683 |
| M-CSF | 0.0003 | 0.736 |
| IL-10 | 0.0033 | 0.749 |
| IL-18 | 0.0017 | 0.754 |
| IL-1RA | 0.0001 | 0.882 |
| HGF | 0.0000 | 0.907 |
| CXCL9 | 0.0270 | 0.920 |
| IL-6 | 0.0000 | 1.129 |
| CXCL10 | 0.0024 | 1.179 |
| CCL7 | 0.0161 | 1.287 |
| CCL2 | 0.0003 | 1.510 |

COVID-CNS: NeuroCOVID/COVID

| Protein | p_value | log2FC |
|---|---|---|
| IL-3 | 0.0123 | -0.9919 |
| IL-18 | 0.0007 | -0.8797 |
| PDGFbb | 0.0021 | -0.7306 |
| IL-6 | 0.0043 | 0.7541 |

**b**

| ID | Description | Ratio | pvalue | Count |
|---|---|---|---|---|
| hsa04060 | Cytokine-cytokine receptor interaction | 15/17 | 1.36E-20 | 15 |
| hsa04061 | Viral protein interaction with cytokine and cytokine receptor | 11/17 | 4.15E-18 | 11 |
| hsa05144 | Malaria | 7/17 | 3.01E-12 | 7 |
| hsa05323 | Rheumatoid arthritis | 7/17 | 2.73E-10 | 7 |
| hsa05142 | Chagas disease | 7/17 | 5.28E-10 | 7 |
| hsa05143 | African trypanosomiasis | 5/17 | 7.18E-09 | 5 |
| hsa04657 | IL-17 signaling pathway | 6/17 | 1.79E-08 | 6 |
| hsa05164 | Influenza A | 7/17 | 1.99E-08 | 7 |
| hsa04620 | Toll-like receptor signaling pathway | 6/17 | 3.3E-08 | 6 |
| hsa05321 | Inflammatory bowel disease | 5/17 | 1.32E-07 | 5 |
| hsa04062 | Chemokine signaling pathway | 6/17 | 1.27E-06 | 6 |
| hsa05146 | Amoebiasis | 5/17 | 1.27E-06 | 5 |
| hsa05417 | Lipid and atherosclerosis | 6/17 | 2.45E-06 | 6 |
| hsa05134 | Legionellosis | 4/17 | 4.12E-06 | 4 |
| hsa05135 | Yersinia infection | 5/17 | 5.46E-06 | 5 |
| hsa05133 | Pertussis | 4/17 | 1.31E-05 | 4 |
| hsa04630 | JAK-STAT signaling pathway | 5/17 | 1.4E-05 | 5 |
| hsa05152 | Tuberculosis | 5/17 | 2.07E-05 | 5 |
| hsa05330 | Allograft rejection | 3/17 | 5.43E-05 | 3 |
| hsa04668 | TNF signaling pathway | 4/17 | 6.49E-05 | 4 |
| hsa05171 | Coronavirus disease - COVID-19 | 5/17 | 7.00E-05 | 5 |

**Supplementary Fig. 3: Pathways enriched during neurological complications of COVID-19**

(a) Cytokine differences in ISARIC Abnormal GCS and COVID-CNS neuro-COVID groups (positive or negative fold change denoted in far right column, significant by two-tailed Mann-Whitney tests

(b) KEGG enrichment pathway analysis of the ISARIC cohort (GCS≤14/GCS=15; pathways significant in both ISARIC and COVID-CNS cohorts shaded in grey)

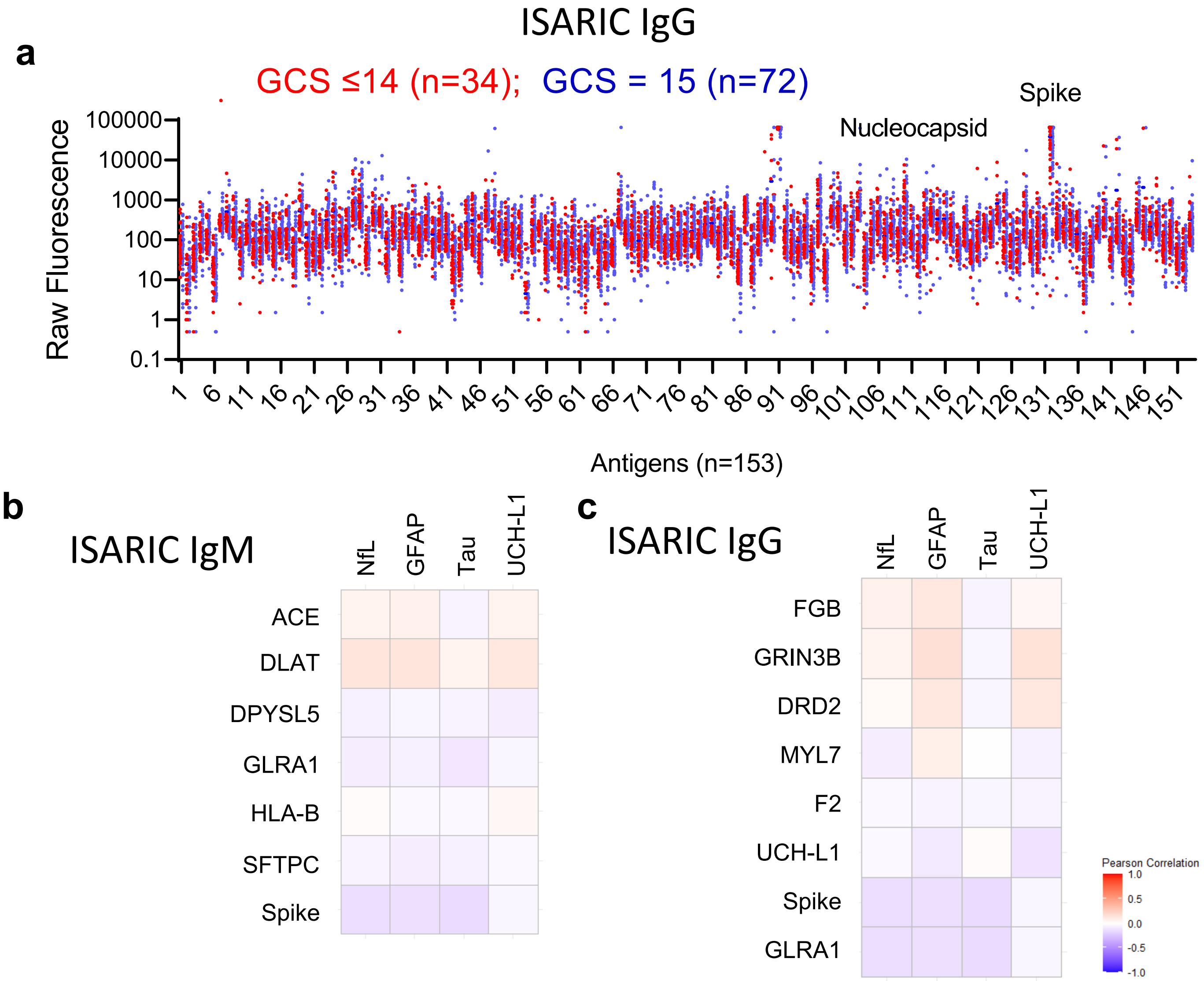


**Supplementary Fig. 4: ISARIC (acute) autoantibody screen**

(a) Raw fluorescence data from the protein chip used to screen antibodies against 153 antigens.

(b) Frequency of IgM autoantibodies in the ISARIC (acute) cohort (*Fisher's exact tests p<0.05)

(c) Heatmaps showing the insignificant Pearson's correlations between IgM or IgG autoantibodies and brain injury markers, UCH-L1, Tau, GFAP, and NfL

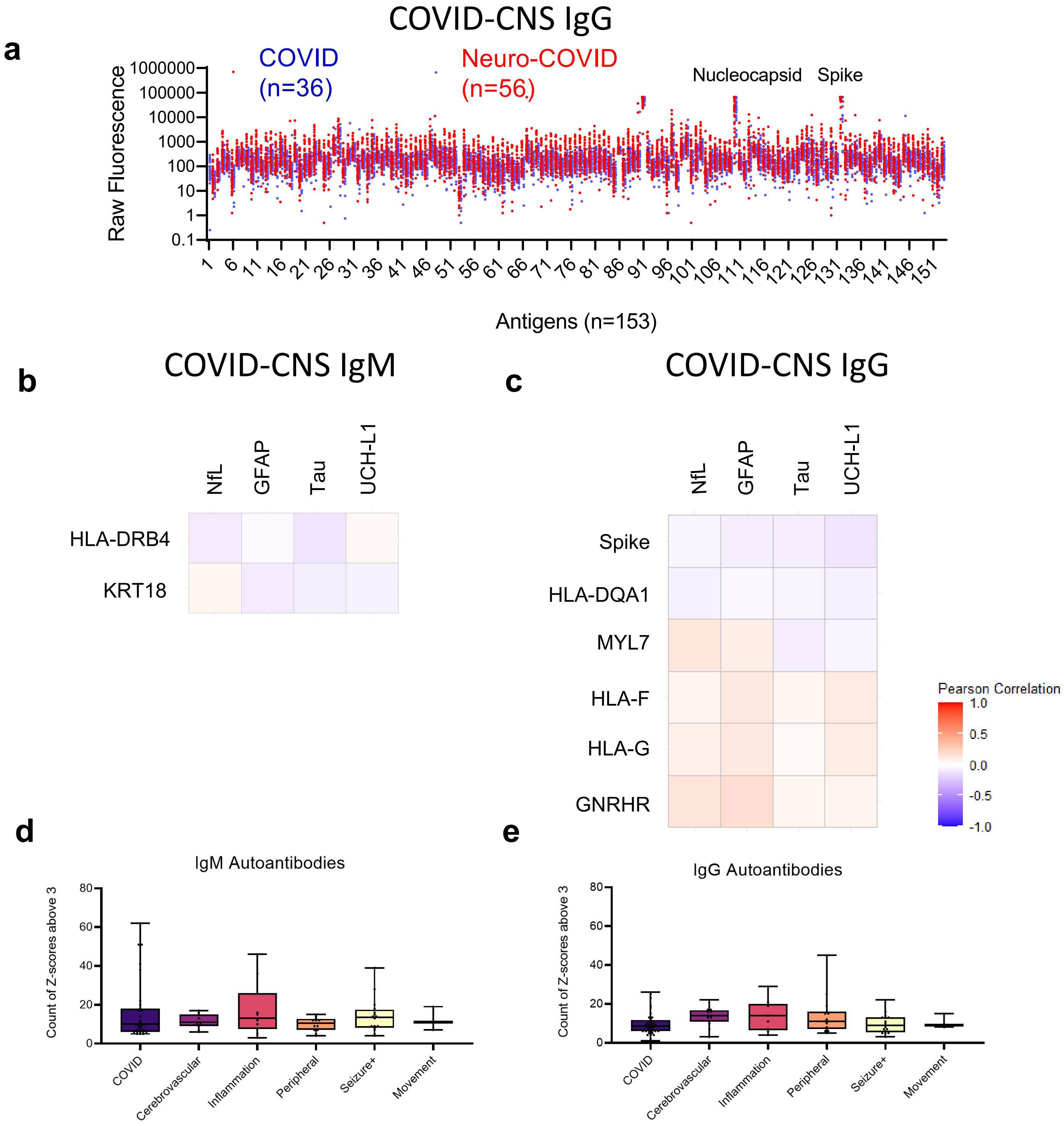


**Supplementary Fig 5: COVID-CNS (early and late convalescent) autoantibody screen**

(a) Raw fluorescence data from the protein chip used to screen antibodies against 153 antigens . The raw fluorescence shows higher binding in the Neuro-COVID compared with COVID, but this difference is not found after normalization (see Figure 3,4).

(b) Heatmaps showing the insignificant Pearson's correlations between IgM or (c) IgG autoantibodies and brain injury markers, UCH-L1, Tau, GFAP, and NfL

(d) Count of IgM and (e) IgG autoantibody positive hits split by condition (Z-scores above 3)

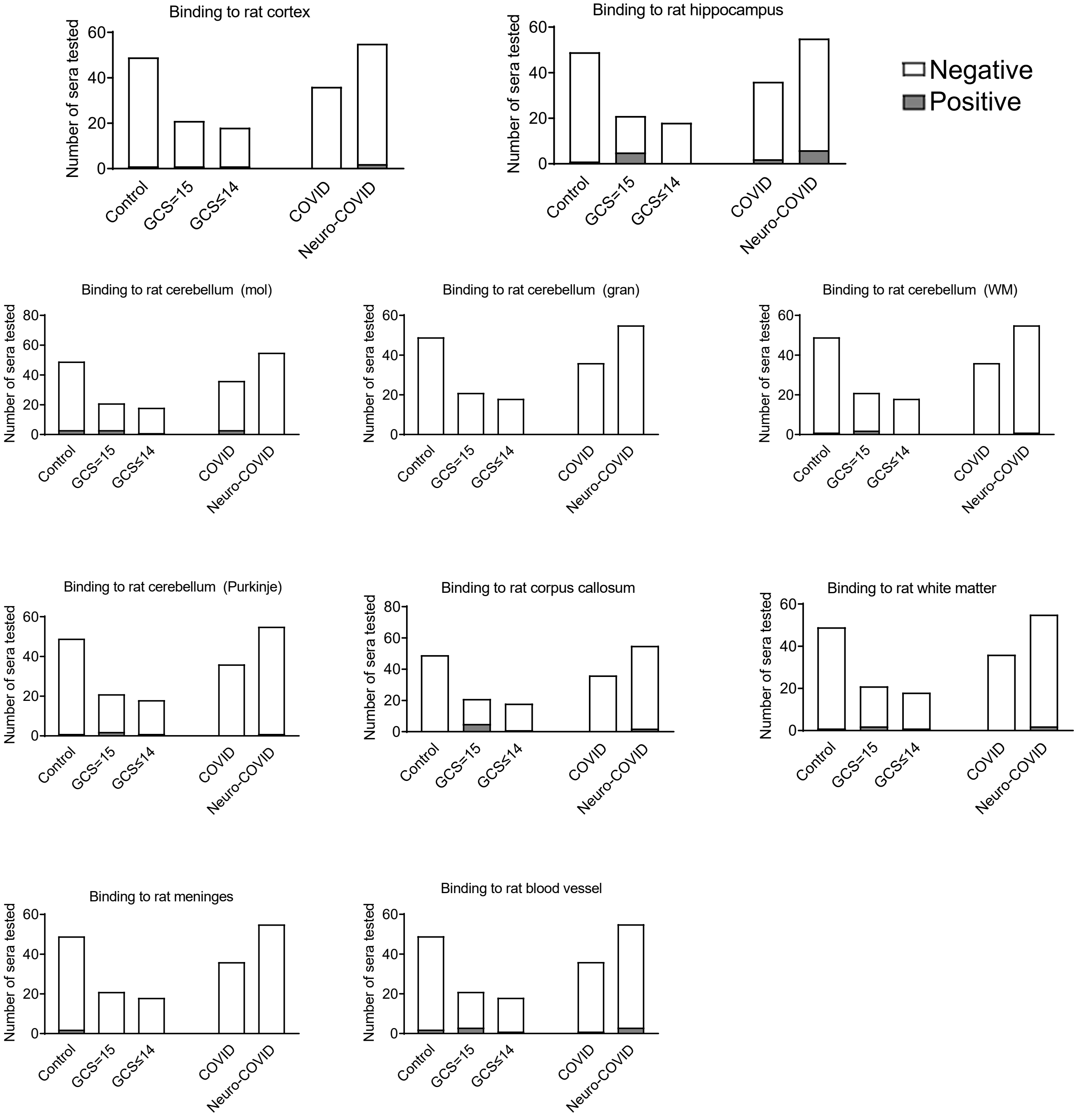


**Supplementary Fig. 6: CNS antibody screen**

Rat brain reactivity as detected by IHC in sera from ISARIC and COVID-CNS cohorts. The numbers positive were analysed by Fisher's exact test with Benjamini and Hochberg correction. Only binding to brainstem showed significant differences between groups, as shown in main text Fig. 3.

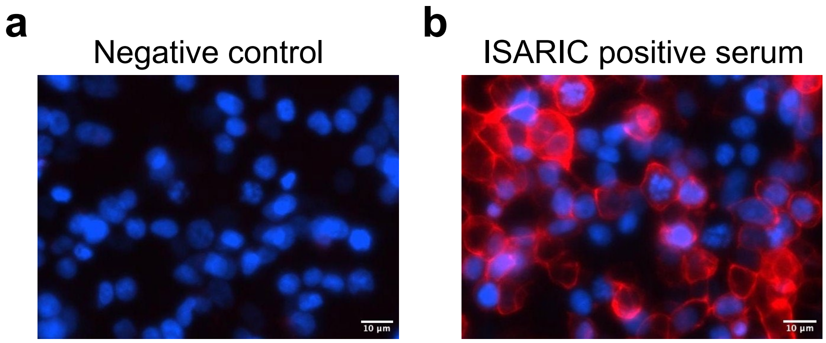


**Supplementary Fig. 7: CNS autoantibody screen by cell-based assay**
Serum samples selected from the rat brain reactive screen were checked for specific autoantibodies (LGI1, CASPR2, NMDAR, and $GABA_B$ receptor). Shown here is the (a) negative control and the (b) ISARIC cohort participant sample that was positive for $GABA_B$ receptor antibodies
