# Supplementary material for: Para-infectious brain injury in COVID-19 persists at follow-up despite attenuated cytokine and autoantibody responses
Source: Nat Commun. 2023 Dec 22;14:8487. doi: 10.1038/s41467-023-42320-4 (PMC10746705; doi:10.1038/s41467-023-42320-4)
Supplement: Supplementary file 3 — Reporting Summary [file 41467_2023_42320_MOESM3_ESM.pdf]

nature portfolio

Corresponding author(s): Benedict D. Michael

Last updated by author(s): 03/10/2023

Reporting Summary

Nature Portfolio wishes to improve the reproducibility of the work that we publish. This form provides structure for consistency and transparency in reporting. For further information on Nature Portfolio policies, see our Editorial Policies and the Editorial Policy Checklist.

Statistics

For all statistical analyses, confirm that the following items are present in the figure legend, table legend, main text, or Methods section.

n/a	Confirmed	
☐	☒	The exact sample size (*n*) for each experimental group/condition, given as a discrete number and unit of measurement
☐	☒	A statement on whether measurements were taken from distinct samples or whether the same sample was measured repeatedly
☐	☒	The statistical test(s) used AND whether they are one- or two-sided<br>*Only common tests should be described solely by name; describe more complex techniques in the Methods section.*
☐	☒	A description of all covariates tested
☐	☒	A description of any assumptions or corrections, such as tests of normality and adjustment for multiple comparisons
☐	☒	A full description of the statistical parameters including central tendency (e.g. means) or other basic estimates (e.g. regression coefficient) AND variation (e.g. standard deviation) or associated estimates of uncertainty (e.g. confidence intervals)
☐	☒	For null hypothesis testing, the test statistic (e.g. *F*, *t*, *r*) with confidence intervals, effect sizes, degrees of freedom and *P* value noted<br>*Give P values as exact values whenever suitable.*
☒	☐	For Bayesian analysis, information on the choice of priors and Markov chain Monte Carlo settings
☒	☐	For hierarchical and complex designs, identification of the appropriate level for tests and full reporting of outcomes
☐	☒	Estimates of effect sizes (e.g. Cohen's *d*, Pearson's *r*), indicating how they were calculated

Our web collection on statistics for biologists contains articles on many of the points above.

Software and code

Policy information about availability of computer code

Data collection	Quanterix HD-X software was used for brain injury marker data collection. Bio-Rad software was used for luminex cytokine data collection. Data for autoantibody fluorescence by HuProt microarray was collected using GenePix Pro v4 software.
Data analysis	Prism software (version 9.4.1, Graph Pad Software Inc.) was used for graph generation and statistical analysis. Heatmaps, volcano plots and Chord diagrams were made using R studio (version 4.1.1 RStudio, PBC). The 2D cytokine network analyses were created using the qgraph package in R software. Proteins which were statistically significantly different in the COVID positive controls (GCS=15 or COVID groups, respectively) versus the GCS less than or equal to 14 or neurological cases were analysed with the KEGG (Kyoto Encyclopedia of Genes and Genomes) database.

For manuscripts utilizing custom algorithms or software that are central to the research but not yet described in published literature, software must be made available to editors and reviewers. We strongly encourage code deposition in a community repository (e.g. GitHub). See the Nature Portfolio guidelines for submitting code & software for further information.

Data

Policy information about availability of data

All manuscripts must include a data availability statement. This statement should provide the following information, where applicable:

- Accession codes, unique identifiers, or web links for publicly available datasets
- A description of any restrictions on data availability
- For clinical datasets or third party data, please ensure that the statement adheres to our policy

The individual-level data from these studies is thus not publicly available to maintain confidentiality. Data generated by the ISARIC4C consortium is available for collaborative analysis projects through an independent data and materials access committee at isaric4c.net/sample_access. Data and samples from the COVID-Clinical Neuroscience Study are available through collaborative research by application through the NIHR bioresource at https://bioresource.nihr.ac.uk/using-our-bioresource/apply-for-bioresource-data-access/. Brain injury marker and immune mediator data are present in the paper and in the source data file.

Research involving human participants, their data, or biological material

Policy information about studies with human participants or human data. See also policy information about sex, gender (identity/presentation), and sexual orientation and race, ethnicity and racism.

Reporting on sex and gender	Sex was self-reported. Differences based on these characteristics were not analyzed.
Reporting on race, ethnicity, or other socially relevant groupings	Differences based on these characteristics were not analyzed.
Population characteristics	Healthy controls: Median age=S0 (35% male); COVID: Median age=52(58% male); Neuro-COVID: Median age=61 (64% male)
Recruitment	ISARIC participants were recruited prospectively during their hospitalization with COVID-19. Healthy controls volunteered to be a part of the NIHR Bi ore source biobank prior to the COVID-19 pandemic. COVID-Clinical Neuroscience Study participants were recruited during hospitalisation or after discharge. There is the potential for self-selection bias as this was a voluntary research study. The potential impact could be that less severe COVID-19 and/or neurology patients participate in the study.
Ethics oversight	Ethical approval for CCP-UK was given by the South Central - Oxford C Research Ethics Committee in England (Ref 13/SC/0149) and the Scotland A Research Ethics Committee (Ref 20/SS/0028).<br>Participants were recruited into the COVID-Clinical Neuroscience Study (COVID-CNS) and either the participant or their next of kin consented in accordance with the ethically-approved NIHR Bioresource, (East of England—Cambridge Central Research Ethics Committee (Ref Ref 17/EE/0025; 22/EE/0230).

Note that full information on the approval of the study protocol must also be provided in the manuscript.

Field-specific reporting

Please select the one below that is the best fit for your research. If you are not sure, read the appropriate sections before making your selection.

☒ Life sciences ☐ Behavioural & social sciences ☐ Ecological, evolutionary & environmental sciences

For a reference copy of the document with all sections, see nature.com/documents/nr-reporting-summary-flat.pdf

Life sciences study design

All studies must disclose on these points even when the disclosure is negative.

Sample size	Based on earlier studies of brain injury markers and cytokines following COVID-19, we calculated that the sample sized required would be 26 patients/group for 95% power to detect a significant difference in mediator abundance at p<0.01.
Data exclusions	None. There are a few values that missing due to no detection by Simoa and HuProt Microarray.
Replication	None.
Randomization	N/A. The ISARIC study was prospective and the COVID-Clinical Neuroscience Study was retrospective. The participants were not assigned to any different groups.
Blinding	Blinding was not performed with participants in these studies, however, serum samples were analysed with de-identified numeric identification numbers that the researchers did not know what group they belonged to when assays were run.

Reporting for specific materials, systems and methods

We require information from authors about some types of materials, experimental systems and methods used in many studies. Here, indicate whether each material, system or method listed is relevant to your study. If you are not sure if a list item applies to your research, read the appropriate section before selecting a response.

Materials & experimental systems

n/a	Involved in the study
☐	☒ Antibodies
☐	☒ Eukaryotic cell lines
☒	☐ Palaeontology and archaeology
☒	☐ Animals and other organisms
☒	☐ Clinical data
☒	☐ Dual use research of concern
☒	☐ Plants

Methods

n/a	Involved in the study
☒	☐ ChIP-seq
☒	☐ Flow cytometry
☒	☐ MRI-based neuroimaging

Antibodies

Antibodies used

Quanterix Simoa Neurology 4-plex B for HD-X Cat#103345; Bio-rad Human 48 cytokine plex Cat#12007283

Validation

Proprietary antibodies validated by Manufacturer's
https://www.quanterix.com/simoa-assay-kits/neuro-4-plex-b-nf-l-gfap-uch-l1-tau/
https://www.bio-rad.com/en-uk/sku/12007283-bio-plex-pro-human-cytokine-screening-panel-48-plex?ID=12007283

Eukaryotic cell lines

Policy information about cell lines and Sex and Gender in Research

Cell line source(s)

HEK 293T was originally purchased from ATCC.

Authentication

The cell line was as an authenticated cell line and were not independently validated.

Mycoplasma contamination

The cell lines were periodically tested for mycoplasma contamination since passaging after purchase.

Commonly misidentified lines
(See ICLAC register)

None used.
